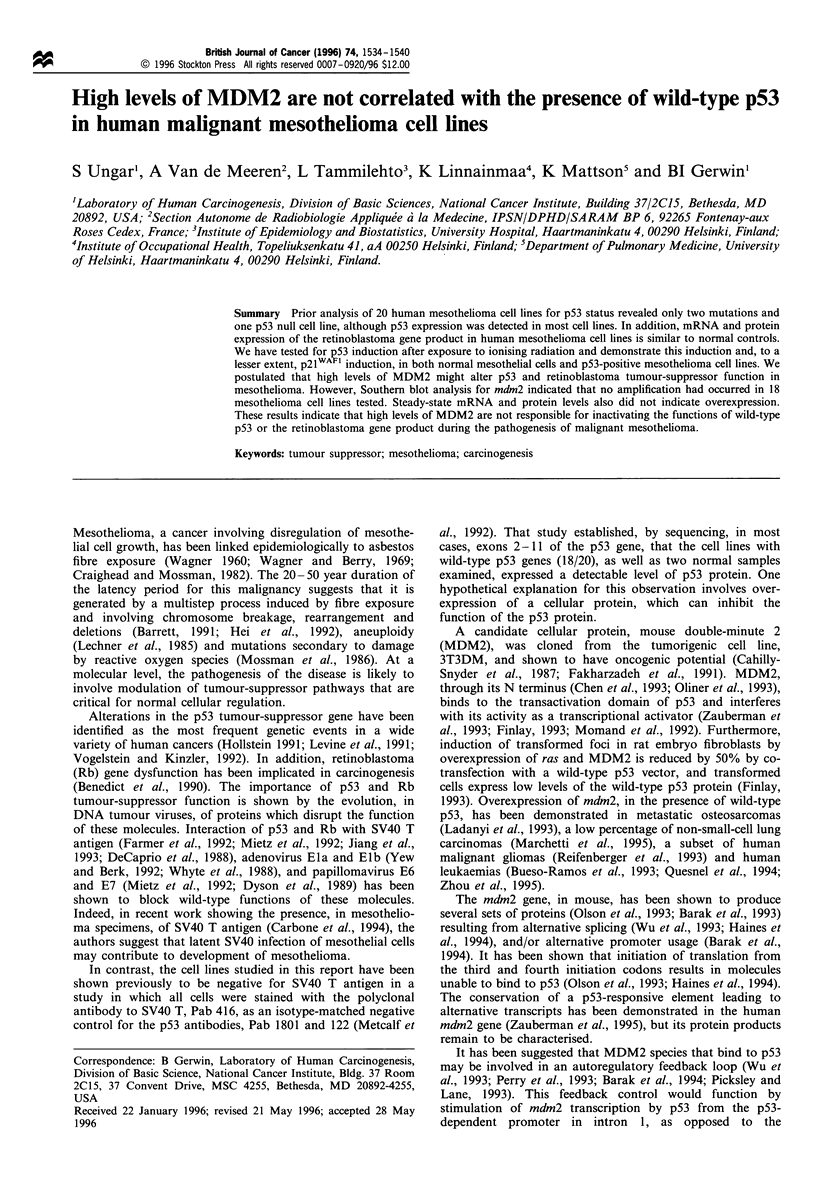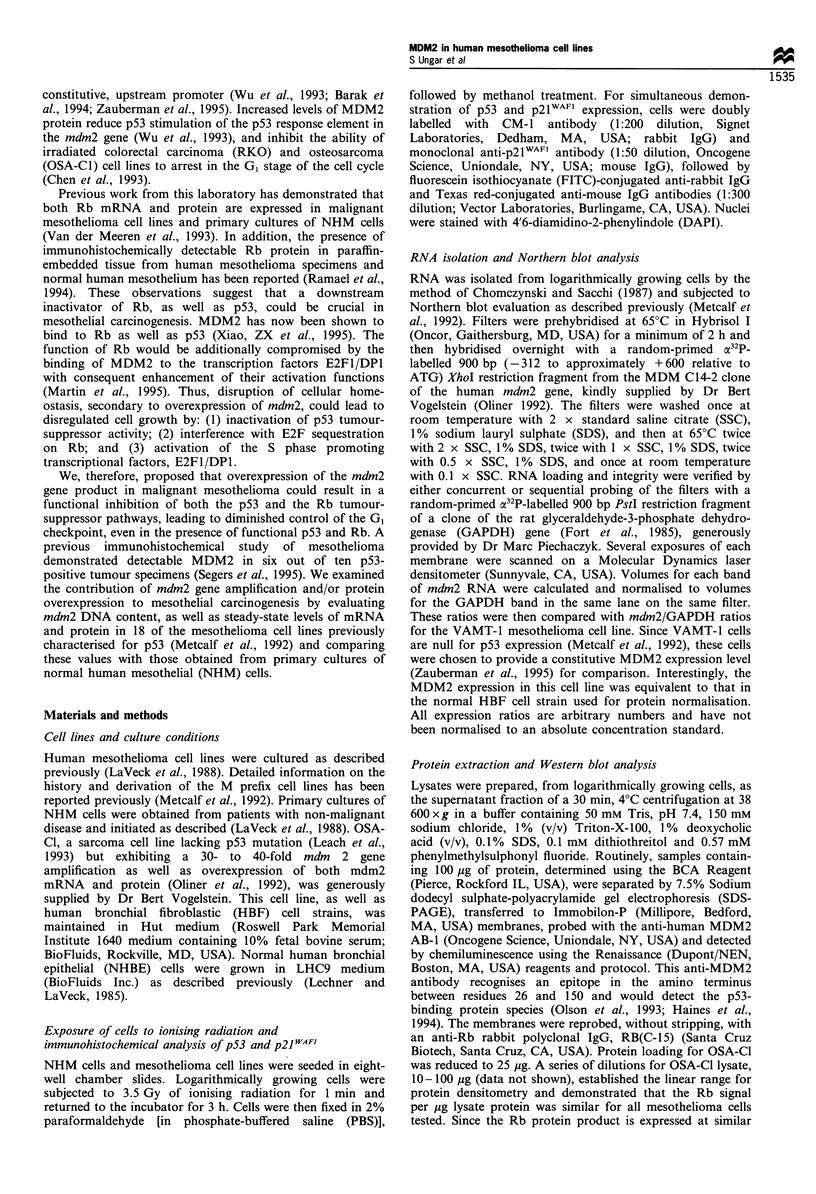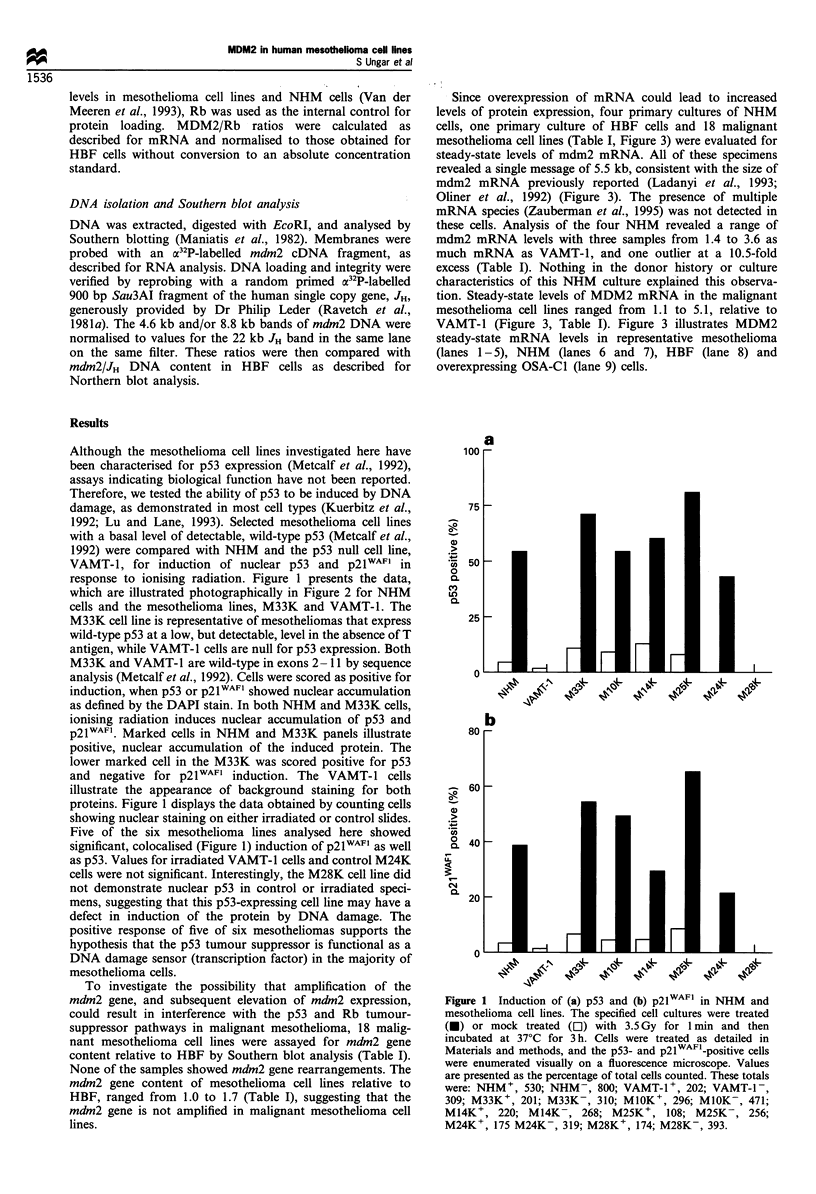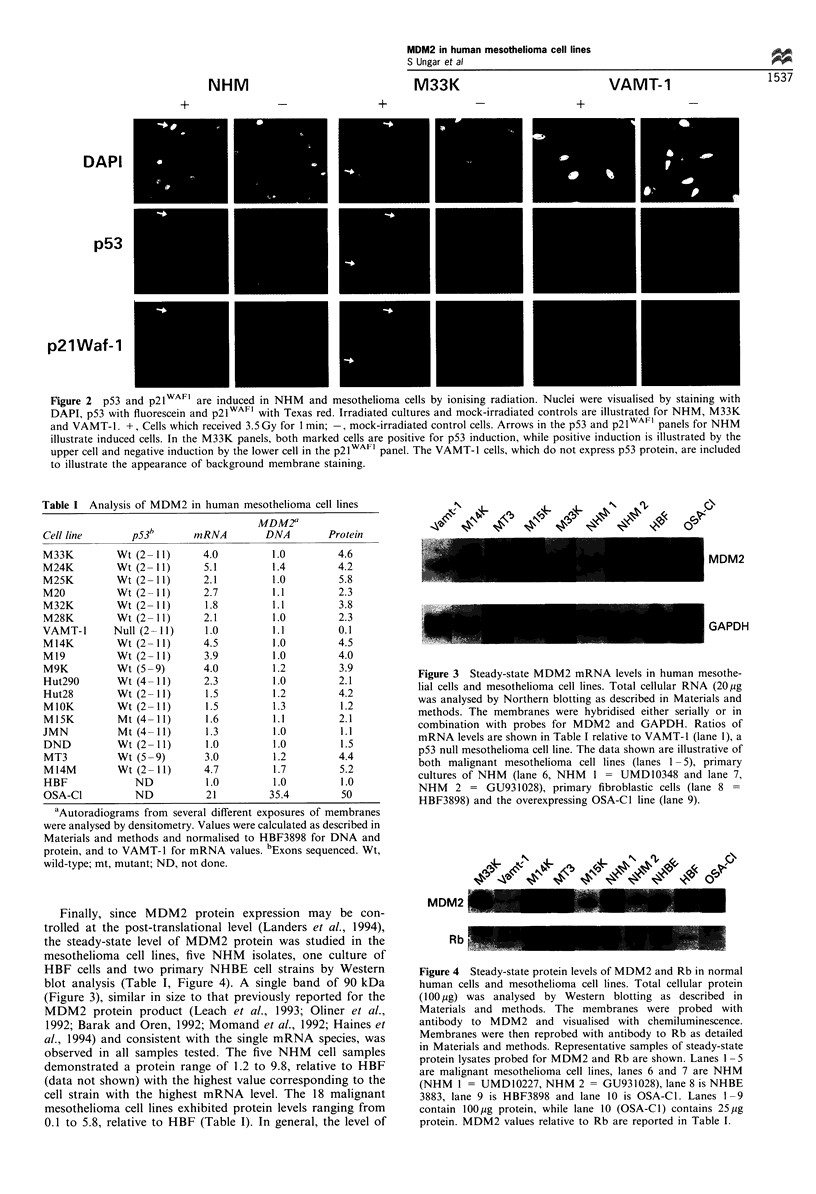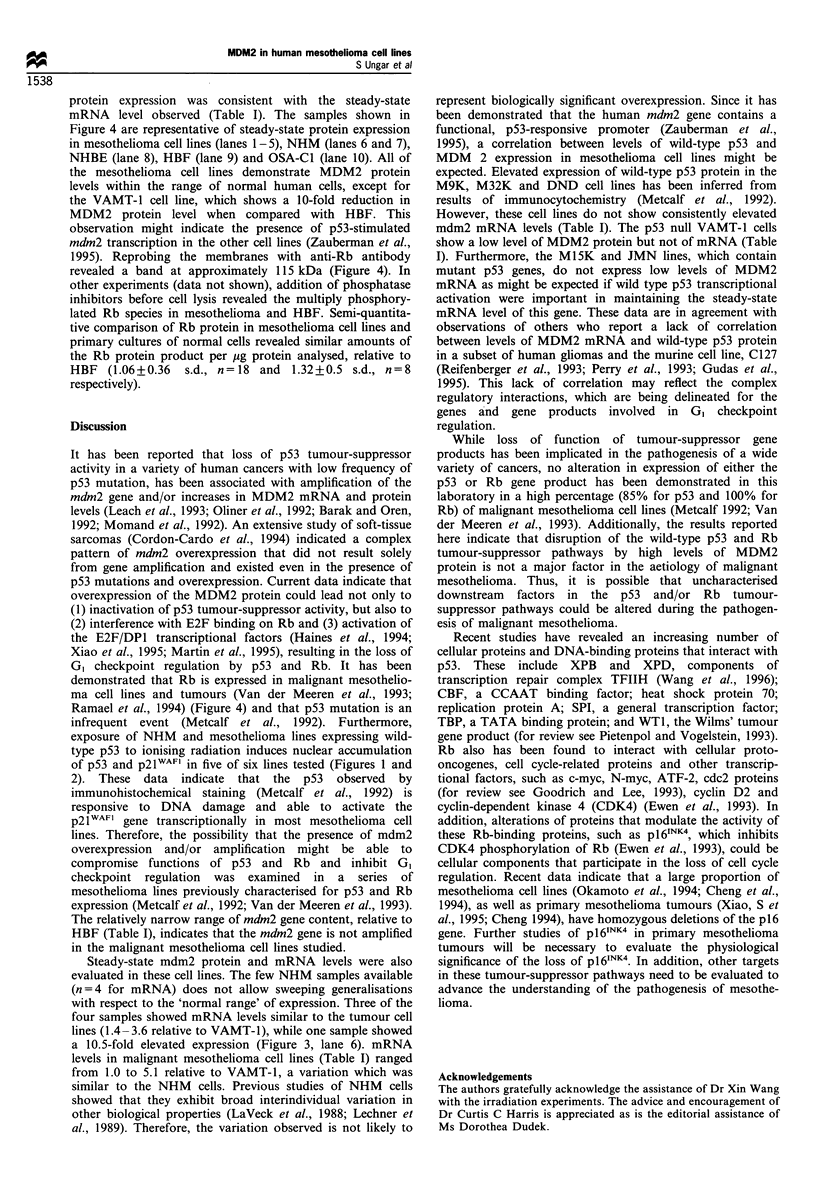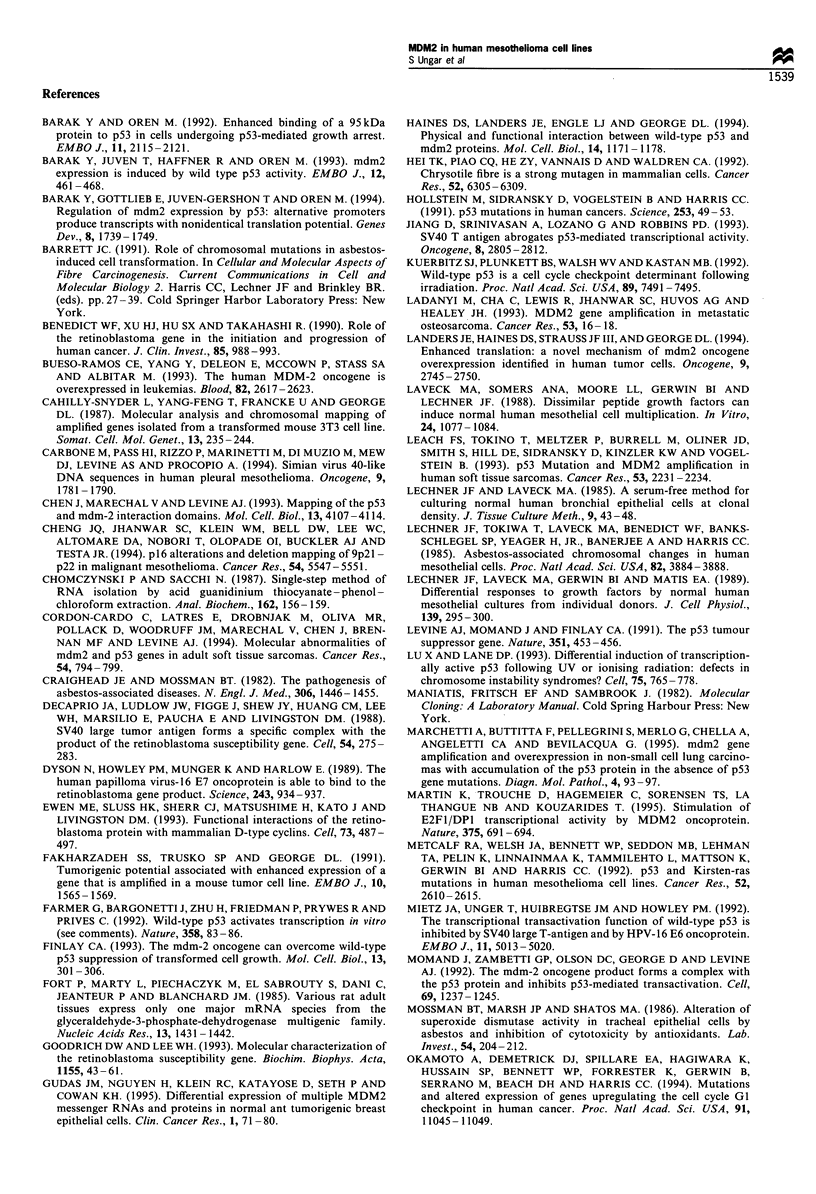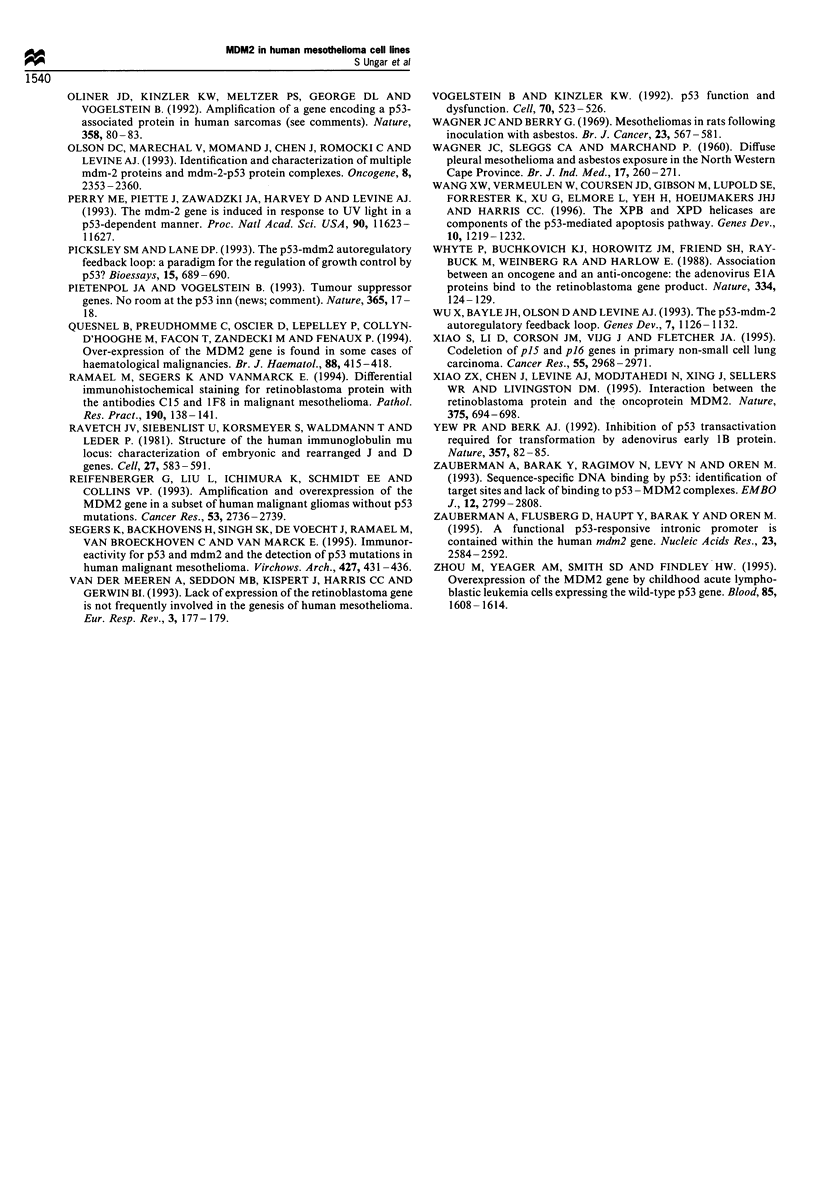# High levels of MDM2 are not correlated with the presence of wild-type p53 in human malignant mesothelioma cell lines.

**DOI:** 10.1038/bjc.1996.585

**Published:** 1996-11

**Authors:** S. Ungar, A. Van de Meeren, L. Tammilehto, K. Linnainmaa, K. Mattson, B. I. Gerwin

**Affiliations:** Laboratory of Human Carcinogenesis, Division of Basic Sciences, National Cancer Institute, Bethesda, MD 20892, USA.

## Abstract

**Images:**


British Journal of Cancer (1996) 74, 1534-1540
© 1996 Stockton Press All rights reserved 0007-0920/96 $12.00

### High levels of MDM2 are not correlated with the presence of wild-type p53 in human malignant mesothelioma cell lines

S Ungar[1], A Van de Meeren[2], L Tammilehto[3], K Linnainmaa[4], K Mattson[5] and BI Gerwin[1]

[1]*Laboratory of Human Carcinogenesis, Division of Basic Sciences, National Cancer Institute, Building 37/2C15, Bethesda, MD 20892, USA;* [2]*Section Autonome de Radiobiologie Appliquée à la Medecine, IPSN/DPHD/SARAM BP 6, 92265 Fontenay-aux Roses Cedex, France;* [3]*Institute of Epidemiology and Biostatistics, University Hospital, Haartmaninkatu 4, 00290 Helsinki, Finland;* [4]*Institute of Occupational Health, Topeliuksenkatu 41, aA 00250 Helsinki, Finland;* [5]*Department of Pulmonary Medicine, University of Helsinki, Haartmaninkatu 4, 00290 Helsinki, Finland.*

**Summary** Prior analysis of 20 human mesothelioma cell lines for p53 status revealed only two mutations and one p53 null cell line, although p53 expression was detected in most cell lines. In addition, mRNA and protein expression of the retinoblastoma gene product in human mesothelioma cell lines is similar to normal controls. We have tested for p53 induction after exposure to ionising radiation and demonstrate this induction and, to a lesser extent, $p21^{WAF1}$ induction, in both normal mesothelial cells and p53-positive mesothelioma cell lines. We postulated that high levels of MDM2 might alter p53 and retinoblastoma tumour-suppressor function in mesothelioma. However, Southern blot analysis for *mdm2* indicated that no amplification had occurred in 18 mesothelioma cell lines tested. Steady-state mRNA and protein levels also did not indicate overexpression. These results indicate that high levels of MDM2 are not responsible for inactivating the functions of wild-type p53 or the retinoblastoma gene product during the pathogenesis of malignant mesothelioma.

**Keywords:** tumour suppressor; mesothelioma; carcinogenesis

Mesothelioma, a cancer involving disregulation of mesothelial cell growth, has been linked epidemiologically to asbestos fibre exposure (Wagner 1960; Wagner and Berry, 1969; Craighead and Mossman, 1982). The 20-50 year duration of the latency period for this malignancy suggests that it is generated by a multistep process induced by fibre exposure and involving chromosome breakage, rearrangement and deletions (Barrett, 1991; Hei *et al.*, 1992), aneuploidy (Lechner *et al.*, 1985) and mutations secondary to damage by reactive oxygen species (Mossman *et al.*, 1986). At a molecular level, the pathogenesis of the disease is likely to involve modulation of tumour-suppressor pathways that are critical for normal cellular regulation.

Alterations in the p53 tumour-suppressor gene have been identified as the most frequent genetic events in a wide variety of human cancers (Hollstein 1991; Levine *et al.*, 1991; Vogelstein and Kinzler, 1992). In addition, retinoblastoma (Rb) gene dysfunction has been implicated in carcinogenesis (Benedict *et al.*, 1990). The importance of p53 and Rb tumour-suppressor function is shown by the evolution, in DNA tumour viruses, of proteins which disrupt the function of these molecules. Interaction of p53 and Rb with SV40 T antigen (Farmer *et al.*, 1992; Mietz *et al.*, 1992; Jiang *et al.*, 1993; DeCaprio *et al.*, 1988), adenovirus Ela and Elb (Yew and Berk, 1992; Whyte *et al.*, 1988), and papillomavirus E6 and E7 (Mietz *et al.*, 1992; Dyson *et al.*, 1989) has been shown to block wild-type functions of these molecules. Indeed, in recent work showing the presence, in mesothelioma specimens, of SV40 T antigen (Carbone *et al.*, 1994), the authors suggest that latent SV40 infection of mesothelial cells may contribute to development of mesothelioma.

In contrast, the cell lines studied in this report have been shown previously to be negative for SV40 T antigen in a study in which all cells were stained with the polyclonal antibody to SV40 T, Pab 416, as an isotype-matched negative control for the p53 antibodies, Pab 1801 and 122 (Metcalf *et al.*, 1992). That study established, by sequencing, in most cases, exons 2-11 of the p53 gene, that the cell lines with wild-type p53 genes (18/20), as well as two normal samples examined, expressed a detectable level of p53 protein. One hypothetical explanation for this observation involves overexpression of a cellular protein, which can inhibit the function of the p53 protein.

A candidate cellular protein, mouse double-minute 2 (MDM2), was cloned from the tumorigenic cell line, 3T3DM, and shown to have oncogenic potential (Cahilly-Snyder *et al.*, 1987; Fakharzadeh *et al.*, 1991). MDM2, through its N terminus (Chen *et al.*, 1993; Oliner *et al.*, 1993), binds to the transactivation domain of p53 and interferes with its activity as a transcriptional activator (Zauberman *et al.*, 1993; Finlay, 1993; Momand *et al.*, 1992). Furthermore, induction of transformed foci in rat embryo fibroblasts by overexpression of *ras* and MDM2 is reduced by 50% by co-transfection with a wild-type p53 vector, and transformed cells express low levels of the wild-type p53 protein (Finlay, 1993). Overexpression of *mdm2*, in the presence of wild-type p53, has been demonstrated in metastatic osteosarcomas (Ladanyi *et al.*, 1993), a low percentage of non-small-cell lung carcinomas (Marchetti *et al.*, 1995), a subset of human malignant gliomas (Reifenberger *et al.*, 1993) and human leukaemias (Bueso-Ramos *et al.*, 1993; Quesnel *et al.*, 1994; Zhou *et al.*, 1995).

The *mdm2* gene, in mouse, has been shown to produce several sets of proteins (Olson *et al.*, 1993; Barak *et al.*, 1993) resulting from alternative splicing (Wu *et al.*, 1993; Haines *et al.*, 1994), and/or alternative promoter usage (Barak *et al.*, 1994). It has been shown that initiation of translation from the third and fourth initiation codons results in molecules unable to bind to p53 (Olson *et al.*, 1993; Haines *et al.*, 1994). The conservation of a p53-responsive element leading to alternative transcripts has been demonstrated in the human *mdm2* gene (Zauberman *et al.*, 1995), but its protein products remain to be characterised.

It has been suggested that MDM2 species that bind to p53 may be involved in an autoregulatory feedback loop (Wu *et al.*, 1993; Perry *et al.*, 1993; Barak *et al.*, 1994; Picksley and Lane, 1993). This feedback control would function by stimulation of *mdm2* transcription by p53 from the p53-dependent promoter in intron 1, as opposed to the

Correspondence: B Gerwin, Laboratory of Human Carcinogenesis, Division of Basic Science, National Cancer Institute, Bldg. 37 Room 2C15, 37 Convent Drive, MSC 4255, Bethesda, MD 20892-4255, USA
Received 22 January 1996; revised 21 May 1996; accepted 28 May 1996

constitutive, upstream promoter (Wu et al., 1993; Barak et al., 1994; Zauberman et al., 1995). Increased levels of MDM2 protein reduce p53 stimulation of the p53 response element in the *mdm2* gene (Wu et al., 1993), and inhibit the ability of irradiated colorectal carcinoma (RKO) and osteosarcoma (OSA-C1) cell lines to arrest in the $G_1$ stage of the cell cycle (Chen et al., 1993).

Previous work from this laboratory has demonstrated that both Rb mRNA and protein are expressed in malignant mesothelioma cell lines and primary cultures of NHM cells (Van der Meeren et al., 1993). In addition, the presence of immunohistochemically detectable Rb protein in paraffin-embedded tissue from human mesothelioma specimens and normal human mesothelium has been reported (Ramael et al., 1994). These observations suggest that a downstream inactivator of Rb, as well as p53, could be crucial in mesothelial carcinogenesis. MDM2 has now been shown to bind to Rb as well as p53 (Xiao, ZX et al., 1995). The function of Rb would be additionally compromised by the binding of MDM2 to the transcription factors E2F1/DP1 with consequent enhancement of their activation functions (Martin et al., 1995). Thus, disruption of cellular homeostasis, secondary to overexpression of *mdm2*, could lead to disregulated cell growth by: (1) inactivation of p53 tumour-suppressor activity; (2) interference with E2F sequestration on Rb; and (3) activation of the S phase promoting transcriptional factors, E2F1/DP1.

We, therefore, proposed that overexpression of the *mdm2* gene product in malignant mesothelioma could result in a functional inhibition of both the p53 and the Rb tumour-suppressor pathways, leading to diminished control of the $G_1$ checkpoint, even in the presence of functional p53 and Rb. A previous immunohistochemical study of mesothelioma demonstrated detectable MDM2 in six out of ten p53-positive tumour specimens (Segers et al., 1995). We examined the contribution of *mdm2* gene amplification and/or protein overexpression to mesothelial carcinogenesis by evaluating *mdm2* DNA content, as well as steady-state levels of mRNA and protein in 18 of the mesothelioma cell lines previously characterised for p53 (Metcalf et al., 1992) and comparing these values with those obtained from primary cultures of normal human mesothelial (NHM) cells.

### Materials and methods

### *Cell lines and culture conditions*

Human mesothelioma cell lines were cultured as described previously (LaVeck et al., 1988). Detailed information on the history and derivation of the M prefix cell lines has been reported previously (Metcalf et al., 1992). Primary cultures of NHM cells were obtained from patients with non-malignant disease and initiated as described (LaVeck et al., 1988). OSA-Cl, a sarcoma cell line lacking p53 mutation (Leach et al., 1993) but exhibiting a 30- to 40-fold *mdm* 2 gene amplification as well as overexpression of both mdm2 mRNA and protein (Oliner et al., 1992), was generously supplied by Dr Bert Vogelstein. This cell line, as well as human bronchial fibroblastic (HBF) cell strains, was maintained in Hut medium (Roswell Park Memorial Institute 1640 medium containing 10% fetal bovine serum; BioFluids, Rockville, MD, USA). Normal human bronchial epithelial (NHBE) cells were grown in LHC9 medium (BioFluids Inc.) as described previously (Lechner and LaVeck, 1985).

### *Exposure of cells to ionising radiation and immunohistochemical analysis of p53 and p21$^{WAF1}$*

NHM cells and mesothelioma cell lines were seeded in eight-well chamber slides. Logarithmically growing cells were subjected to 3.5 Gy of ionising radiation for 1 min and returned to the incubator for 3 h. Cells were then fixed in 2% paraformaldehyde [in phosphate-buffered saline (PBS)], followed by methanol treatment. For simultaneous demonstration of p53 and p21$^{WAF1}$ expression, cells were doubly labelled with CM-1 antibody (1:200 dilution, Signet Laboratories, Dedham, MA, USA; rabbit IgG) and monoclonal anti-p21$^{WAF1}$ antibody (1:50 dilution, Oncogene Science, Uniondale, NY, USA; mouse IgG), followed by fluorescein isothiocyanate (FITC)-conjugated anti-rabbit IgG and Texas red-conjugated anti-mouse IgG antibodies (1:300 dilution; Vector Laboratories, Burlingame, CA, USA). Nuclei were stained with 4′6-diamidino-2-phenylindole (DAPI).

### *RNA isolation and Northern blot analysis*

RNA was isolated from logarithmically growing cells by the method of Chomczynski and Sacchi (1987) and subjected to Northern blot evaluation as described previously (Metcalf et al., 1992). Filters were prehybridised at 65°C in Hybrisol I (Oncor, Gaithersburg, MD, USA) for a minimum of 2 h and then hybridised overnight with a random-primed $\alpha^{32}P$-labelled 900 bp (−312 to approximately +600 relative to ATG) *Xho*I restriction fragment from the MDM C14-2 clone of the human *mdm2* gene, kindly supplied by Dr Bert Vogelstein (Oliner 1992). The filters were washed once at room temperature with 2 × standard saline citrate (SSC), 1% sodium lauryl sulphate (SDS), and then at 65°C twice with 2 × SSC, 1% SDS, twice with 1 × SSC, 1% SDS, twice with 0.5 × SSC, 1% SDS, and once at room temperature with 0.1 × SSC. RNA loading and integrity were verified by either concurrent or sequential probing of the filters with a random-primed $\alpha^{32}P$-labelled 900 bp *Pst*I restriction fragment of a clone of the rat glyceraldehyde-3-phosphate dehydrogenase (GAPDH) gene (Fort et al., 1985), generously provided by Dr Marc Piechaczyk. Several exposures of each membrane were scanned on a Molecular Dynamics laser densitometer (Sunnyvale, CA, USA). Volumes for each band of *mdm2* RNA were calculated and normalised to volumes for the GAPDH band in the same lane on the same filter. These ratios were then compared with *mdm2*/GAPDH ratios for the VAMT-1 mesothelioma cell line. Since VAMT-1 cells are null for p53 expression (Metcalf et al., 1992), these cells were chosen to provide a constitutive MDM2 expression level (Zauberman et al., 1995) for comparison. Interestingly, the MDM2 expression in this cell line was equivalent to that in the normal HBF cell strain used for protein normalisation. All expression ratios are arbitrary numbers and have not been normalised to an absolute concentration standard.

### *Protein extraction and Western blot analysis*

Lysates were prepared, from logarithmically growing cells, as the supernatant fraction of a 30 min, 4°C centrifugation at 38 600 × *g* in a buffer containing 50 mM Tris, pH 7.4, 150 mM sodium chloride, 1% (v/v) Triton-X-100, 1% deoxycholic acid (v/v), 0.1% SDS, 0.1 mM dithiothreitol and 0.57 mM phenylmethylsulphonyl fluoride. Routinely, samples containing 100 μg of protein, determined using the BCA Reagent (Pierce, Rockford IL, USA), were separated by 7.5% Sodium dodecyl sulphate-polyacrylamide gel electrophoresis (SDS-PAGE), transferred to Immobilon-P (Millipore, Bedford, MA, USA) membranes, probed with the anti-human MDM2 AB-1 (Oncogene Science, Uniondale, NY, USA) and detected by chemiluminescence using the Renaissance (Dupont/NEN, Boston, MA, USA) reagents and protocol. This anti-MDM2 antibody recognises an epitope in the amino terminus between residues 26 and 150 and would detect the p53-binding protein species (Olson et al., 1993; Haines et al., 1994). The membranes were reprobed, without stripping, with an anti-Rb rabbit polyclonal IgG, RB(C-15) (Santa Cruz Biotech, Santa Cruz, CA, USA). Protein loading for OSA-Cl was reduced to 25 μg. A series of dilutions for OSA-Cl lysate, 10−100 μg (data not shown), established the linear range for protein densitometry and demonstrated that the Rb signal per μg lysate protein was similar for all mesothelioma cells tested. Since the Rb protein product is expressed at similar levels in mesothelioma cell lines and NHM cells (Van der Meeren *et al.*, 1993), Rb was used as the internal control for protein loading. MDM2/Rb ratios were calculated as described for mRNA and normalised to those obtained for HBF cells without conversion to an absolute concentration standard.

*DNA isolation and Southern blot analysis*

DNA was extracted, digested with *Eco*RI, and analysed by Southern blotting (Maniatis *et al.*, 1982). Membranes were probed with an $\alpha^{32}$P-labelled *mdm*2 cDNA fragment, as described for RNA analysis. DNA loading and integrity were verified by reprobing with a random primed $\alpha^{32}$P-labelled 900 bp *Sau*3AI fragment of the human single copy gene, $J_H$, generously provided by Dr Philip Leder (Ravetch *et al.*, 1981*a*). The 4.6 kb and/or 8.8 kb bands of *mdm*2 DNA were normalised to values for the 22 kb $J_H$ band in the same lane on the same filter. These ratios were then compared with *mdm*2/$J_H$ DNA content in HBF cells as described for Northern blot analysis.

### Results

Although the mesothelioma cell lines investigated here have been characterised for p53 expression (Metcalf *et al.*, 1992), assays indicating biological function have not been reported. Therefore, we tested the ability of p53 to be induced by DNA damage, as demonstrated in most cell types (Kuerbitz *et al.*, 1992; Lu and Lane, 1993). Selected mesothelioma cell lines with a basal level of detectable, wild-type p53 (Metcalf *et al.*, 1992) were compared with NHM and the p53 null cell line, VAMT-1, for induction of nuclear p53 and p21$^{WAF1}$ in response to ionising radiation. Figure 1 presents the data, which are illustrated photographically in Figure 2 for NHM cells and the mesothelioma lines, M33K and VAMT-1. The M33K cell line is representative of mesotheliomas that express wild-type p53 at a low, but detectable, level in the absence of T antigen, while VAMT-1 cells are null for p53 expression. Both M33K and VAMT-1 are wild-type in exons 2–11 by sequence analysis (Metcalf *et al.*, 1992). Cells were scored as positive for induction, when p53 or p21$^{WAF1}$ showed nuclear accumulation as defined by the DAPI stain. In both NHM and M33K cells, ionising radiation induces nuclear accumulation of p53 and p21$^{WAF1}$. Marked cells in NHM and M33K panels illustrate positive, nuclear accumulation of the induced protein. The lower marked cell in the M33K was scored positive for p53 and negative for p21$^{WAF1}$ induction. The VAMT-1 cells illustrate the appearance of background staining for both proteins. Figure 1 displays the data obtained by counting cells showing nuclear staining on either irradiated or control slides. Five of the six mesothelioma lines analysed here showed significant, colocalised (Figure 1) induction of p21$^{WAF1}$ as well as p53. Values for irradiated VAMT-1 cells and control M24K cells were not significant. Interestingly, the M28K cell line did not demonstrate nuclear p53 in control or irradiated specimens, suggesting that this p53-expressing cell line may have a defect in induction of the protein by DNA damage. The positive response of five of six mesotheliomas supports the hypothesis that the p53 tumour suppressor is functional as a DNA damage sensor (transcription factor) in the majority of mesothelioma cells.

To investigate the possibility that amplification of the *mdm*2 gene, and subsequent elevation of *mdm*2 expression, could result in interference with the p53 and Rb tumour-suppressor pathways in malignant mesothelioma, 18 malignant mesothelioma cell lines were assayed for *mdm*2 gene content relative to HBF by Southern blot analysis (Table I). None of the samples showed *mdm*2 gene rearrangements. The *mdm*2 gene content of mesothelioma cell lines relative to HBF, ranged from 1.0 to 1.7 (Table I), suggesting that the *mdm*2 gene is not amplified in malignant mesothelioma cell lines.

Since overexpression of mRNA could lead to increased levels of protein expression, four primary cultures of NHM cells, one primary culture of HBF cells and 18 malignant mesothelioma cell lines (Table I, Figure 3) were evaluated for steady-state levels of mdm2 mRNA. All of these specimens revealed a single message of 5.5 kb, consistent with the size of mdm2 mRNA previously reported (Ladanyi *et al.*, 1993; Oliner *et al.*, 1992) (Figure 3). The presence of multiple mRNA species (Zauberman *et al.*, 1995) was not detected in these cells. Analysis of the four NHM revealed a range of mdm2 mRNA levels with three samples from 1.4 to 3.6 as much mRNA as VAMT-1, and one outlier at a 10.5-fold excess (Table I). Nothing in the donor history or culture characteristics of this NHM culture explained this observation. Steady-state levels of MDM2 mRNA in the malignant mesothelioma cell lines ranged from 1.1 to 5.1, relative to VAMT-1 (Figure 3, Table I). Figure 3 illustrates MDM2 steady-state mRNA levels in representative mesothelioma (lanes 1–5), NHM (lanes 6 and 7), HBF (lane 8) and overexpressing OSA-C1 (lane 9) cells.

**Figure 1** Induction of (**a**) p53 and (**b**) p21$^{WAF1}$ in NHM and mesothelioma cell lines. The specified cell cultures were treated (■) or mock treated (□) with 3.5 Gy for 1 min and then incubated at 37°C for 3 h. Cells were treated as detailed in Materials and methods, and the p53- and p21$^{WAF1}$-positive cells were enumerated visually on a fluorescence microscope. Values are presented as the percentage of total cells counted. These totals were: NHM$^+$, 530; NHM$^-$, 800; VAMT-1$^+$, 202; VAMT-1$^-$, 309; M33K$^+$, 201; M33K$^-$, 310; M10K$^+$, 296; M10K$^-$, 471; M14K$^+$, 220; M14K$^-$, 268; M25K$^+$, 108; M25K$^-$, 256; M24K$^+$, 175 M24K$^-$, 319; M28K$^+$, 174; M28K$^-$, 393.

**Figure 2** p53 and p21$^{WAF1}$ are induced in NHM and mesothelioma cells by ionising radiation. Nuclei were visualised by staining with DAPI, p53 with fluorescein and p21$^{WAF1}$ with Texas red. Irradiated cultures and mock-irradiated controls are illustrated for NHM, M33K and VAMT-1. +, Cells which received 3.5 Gy for 1 min; −, mock-irradiated control cells. Arrows in the p53 and p21$^{WAF1}$ panels for NHM illustrate induced cells. In the M33K panels, both marked cells are positive for p53 induction, while positive induction is illustrated by the upper cell and negative induction by the lower cell in the p21$^{WAF1}$ panel. The VAMT-1 cells, which do not express p53 protein, are included to illustrate the appearance of background membrane staining.

**Table I** Analysis of MDM2 in human mesothelioma cell lines

| *Cell line* | *p53*[b] | *mRNA* | *MDM2*[a] *DNA* | *Protein* |
|---|---|---|---|---|
| M33K | Wt (2–11) | 4.0 | 1.0 | 4.6 |
| M24K | Wt (2–11) | 5.1 | 1.4 | 4.2 |
| M25K | Wt (2–11) | 2.1 | 1.0 | 5.8 |
| M20 | Wt (2–11) | 2.7 | 1.1 | 2.3 |
| M32K | Wt (2–11) | 1.8 | 1.1 | 3.8 |
| M28K | Wt (2–11) | 2.1 | 1.0 | 2.3 |
| VAMT-1 | Null (2–11) | 1.0 | 1.1 | 0.1 |
| M14K | Wt (2–11) | 4.5 | 1.0 | 4.5 |
| M19 | Wt (2–11) | 3.9 | 1.0 | 4.0 |
| M9K | Wt (5–9) | 4.0 | 1.2 | 3.9 |
| Hut290 | Wt (4–11) | 2.3 | 1.0 | 2.1 |
| Hut28 | Wt (2–11) | 1.5 | 1.2 | 4.2 |
| M10K | Wt (2–11) | 1.5 | 1.3 | 1.2 |
| M15K | Mt (4–11) | 1.6 | 1.1 | 2.1 |
| JMN | Mt (4–11) | 1.3 | 1.0 | 1.1 |
| DND | Wt (2–11) | 1.0 | 1.0 | 1.5 |
| MT3 | Wt (5–9) | 3.0 | 1.2 | 4.4 |
| M14M | Wt (2–11) | 4.7 | 1.7 | 5.2 |
| HBF | ND | 1.0 | 1.0 | 1.0 |
| OSA-Cl | ND | 21 | 35.4 | 50 |

[a]Autoradiograms from several different exposures of membranes were analysed by densitometry. Values were calculated as described in Materials and methods and normalised to HBF3898 for DNA and protein, and to VAMT-1 for mRNA values. [b]Exons sequenced. Wt, wild-type; mt, mutant; ND, not done.

Finally, since MDM2 protein expression may be controlled at the post-translational level (Landers *et al.*, 1994), the steady-state level of MDM2 protein was studied in the mesothelioma cell lines, five NHM isolates, one culture of HBF cells and two primary NHBE cell strains by Western blot analysis (Table I, Figure 4). A single band of 90 kDa (Figure 3), similar in size to that previously reported for the MDM2 protein product (Leach *et al.*, 1993; Oliner *et al.*, 1992; Barak and Oren, 1992; Momand *et al.*, 1992; Haines *et al.*, 1994) and consistent with the single mRNA species, was observed in all samples tested. The five NHM cell samples demonstrated a protein range of 1.2 to 9.8, relative to HBF (data not shown) with the highest value corresponding to the cell strain with the highest mRNA level. The 18 malignant mesothelioma cell lines exhibited protein levels ranging from 0.1 to 5.8, relative to HBF (Table I). In general, the level of

**Figure 3** Steady-state MDM2 mRNA levels in human mesothelial cells and mesothelioma cell lines. Total cellular RNA (20 $\mu$g was analysed by Northern blotting as described in Materials and methods. The membranes were hybridised either serially or in combination with probes for MDM2 and GAPDH. Ratios of mRNA levels are shown in Table I relative to VAMT-1 (lane 1), a p53 null mesothelioma cell line. The data shown are illustrative of both malignant mesothelioma cell lines (lanes 1–5), primary cultures of NHM (lane 6, NHM 1 = UMD10348 and lane 7, NHM 2 = GU931028), primary fibroblastic cells (lane 8 = HBF3898) and the overexpressing OSA-Cl line (lane 9).

**Figure 4** Steady-state protein levels of MDM2 and Rb in normal human cells and mesothelioma cell lines. Total cellular protein (100 $\mu$g) was analysed by Western blotting as described in Materials and methods. The membranes were probed with antibody to MDM2 and visualised with chemiluminescence. Membranes were then reprobed with antibody to Rb as detailed in Materials and methods. Representative samples of steady-state protein lysates probed for MDM2 and Rb are shown. Lanes 1–5 are malignant mesothelioma cell lines, lanes 6 and 7 are NHM (NHM 1 = UMD10227, NHM 2 = GU931028), lane 8 is NHBE 3883, lane 9 is HBF3898 and lane 10 is OSA-Cl. Lanes 1–9 contain 100 $\mu$g protein, while lane 10 (OSA-Cl) contains 25 $\mu$g protein. MDM2 values relative to Rb are reported in Table I.

protein expression was consistent with the steady-state mRNA level observed (Table I). The samples shown in Figure 4 are representative of steady-state protein expression in mesothelioma cell lines (lanes 1–5), NHM (lanes 6 and 7), NHBE (lane 8), HBF (lane 9) and OSA-C1 (lane 10). All of the mesothelioma cell lines demonstrate MDM2 protein levels within the range of normal human cells, except for the VAMT-1 cell line, which shows a 10-fold reduction in MDM2 protein level when compared with HBF. This observation might indicate the presence of p53-stimulated *mdm2* transcription in the other cell lines (Zauberman *et al.*, 1995). Reprobing the membranes with anti-Rb antibody revealed a band at approximately 115 kDa (Figure 4). In other experiments (data not shown), addition of phosphatase inhibitors before cell lysis revealed the multiply phosphorylated Rb species in mesothelioma and HBF. Semi-quantitative comparison of Rb protein in mesothelioma cell lines and primary cultures of normal cells revealed similar amounts of the Rb protein product per $\mu g$ protein analysed, relative to HBF ($1.06 \pm 0.36$ s.d., $n = 18$ and $1.32 \pm 0.5$ s.d., $n = 8$ respectively).

### Discussion

It has been reported that loss of p53 tumour-suppressor activity in a variety of human cancers with low frequency of p53 mutation, has been associated with amplification of the *mdm2* gene and/or increases in MDM2 mRNA and protein levels (Leach *et al.*, 1993; Oliner *et al.*, 1992; Barak and Oren, 1992; Momand *et al.*, 1992). An extensive study of soft-tissue sarcomas (Cordon-Cardo *et al.*, 1994) indicated a complex pattern of *mdm2* overexpression that did not result solely from gene amplification and existed even in the presence of p53 mutations and overexpression. Current data indicate that overexpression of the MDM2 protein could lead not only to (1) inactivation of p53 tumour-suppressor activity, but also to (2) interference with E2F binding on Rb and (3) activation of the E2F/DP1 transcriptional factors (Haines *et al.*, 1994; Xiao *et al.*, 1995; Martin *et al.*, 1995), resulting in the loss of $G_1$ checkpoint regulation by p53 and Rb. It has been demonstrated that Rb is expressed in malignant mesothelioma cell lines and tumours (Van der Meeren *et al.*, 1993; Ramael *et al.*, 1994) (Figure 4) and that p53 mutation is an infrequent event (Metcalf *et al.*, 1992). Furthermore, exposure of NHM and mesothelioma lines expressing wild-type p53 to ionising radiation induces nuclear accumulation of p53 and $p21^{WAF1}$ in five of six lines tested (Figures 1 and 2). These data indicate that the p53 observed by immunohistochemical staining (Metcalf *et al.*, 1992) is responsive to DNA damage and able to activate the $p21^{WAF1}$ gene transcriptionally in most mesothelioma cell lines. Therefore, the possibility that the presence of mdm2 overexpression and/or amplification might be able to compromise functions of p53 and Rb and inhibit $G_1$ checkpoint regulation was examined in a series of mesothelioma lines previously characterised for p53 and Rb expression (Metcalf *et al.*, 1992; Van der Meeren *et al.*, 1993). The relatively narrow range of *mdm2* gene content, relative to HBF (Table I), indicates that the *mdm2* gene is not amplified in the malignant mesothelioma cell lines studied.

Steady-state mdm2 protein and mRNA levels were also evaluated in these cell lines. The few NHM samples available ($n = 4$ for mRNA) does not allow sweeping generalisations with respect to the 'normal range' of expression. Three of the four samples showed mRNA levels similar to the tumour cell lines (1.4–3.6 relative to VAMT-1), while one sample showed a 10.5-fold elevated expression (Figure 3, lane 6). mRNA levels in malignant mesothelioma cell lines (Table I) ranged from 1.0 to 5.1 relative to VAMT-1, a variation which was similar to the NHM cells. Previous studies of NHM cells showed that they exhibit broad interindividual variation in other biological properties (LaVeck *et al.*, 1988; Lechner *et al.*, 1989). Therefore, the variation observed is not likely to represent biologically significant overexpression. Since it has been demonstrated that the human *mdm2* gene contains a functional, p53-responsive promoter (Zauberman *et al.*, 1995), a correlation between levels of wild-type p53 and MDM 2 expression in mesothelioma cell lines might be expected. Elevated expression of wild-type p53 protein in the M9K, M32K and DND cell lines has been inferred from results of immunocytochemistry (Metcalf *et al.*, 1992). However, these cell lines do not show consistently elevated mdm2 mRNA levels (Table I). The p53 null VAMT-1 cells show a low level of MDM2 protein but not of mRNA (Table I). Furthermore, the M15K and JMN lines, which contain mutant p53 genes, do not express low levels of MDM2 mRNA as might be expected if wild type p53 transcriptional activation were important in maintaining the steady-state mRNA level of this gene. These data are in agreement with observations of others who report a lack of correlation between levels of MDM2 mRNA and wild-type p53 protein in a subset of human gliomas and the murine cell line, C127 (Reifenberger *et al.*, 1993; Perry *et al.*, 1993; Gudas *et al.*, 1995). This lack of correlation may reflect the complex regulatory interactions, which are being delineated for the genes and gene products involved in $G_1$ checkpoint regulation.

While loss of function of tumour-suppressor gene products has been implicated in the pathogenesis of a wide variety of cancers, no alteration in expression of either the p53 or Rb gene product has been demonstrated in this laboratory in a high percentage (85% for p53 and 100% for Rb) of malignant mesothelioma cell lines (Metcalf 1992; Van der Meeren *et al.*, 1993). Additionally, the results reported here indicate that disruption of the wild-type p53 and Rb tumour-suppressor pathways by high levels of MDM2 protein is not a major factor in the aetiology of malignant mesothelioma. Thus, it is possible that uncharacterised downstream factors in the p53 and/or Rb tumour-suppressor pathways could be altered during the pathogenesis of malignant mesothelioma.

Recent studies have revealed an increasing number of cellular proteins and DNA-binding proteins that interact with p53. These include XPB and XPD, components of transcription repair complex TFIIH (Wang *et al.*, 1996); CBF, a CCAAT binding factor; heat shock protein 70; replication protein A; SPI, a general transcription factor; TBP, a TATA binding protein; and WT1, the Wilms' tumour gene product (for review see Pietenpol and Vogelstein, 1993). Rb also has been found to interact with cellular proto-oncogenes, cell cycle-related proteins and other transcriptional factors, such as c-myc, N-myc, ATF-2, cdc2 proteins (for review see Goodrich and Lee, 1993), cyclin D2 and cyclin-dependent kinase 4 (CDK4) (Ewen *et al.*, 1993). In addition, alterations of proteins that modulate the activity of these Rb-binding proteins, such as $p16^{INK4}$, which inhibits CDK4 phosphorylation of Rb (Ewen *et al.*, 1993), could be cellular components that participate in the loss of cell cycle regulation. Recent data indicate that a large proportion of mesothelioma cell lines (Okamoto *et al.*, 1994; Cheng *et al.*, 1994), as well as primary mesothelioma tumours (Xiao, S *et al.*, 1995; Cheng 1994), have homozygous deletions of the p16 gene. Further studies of $p16^{INK4}$ in primary mesothelioma tumours will be necessary to evaluate the physiological significance of the loss of $p16^{INK4}$. In addition, other targets in these tumour-suppressor pathways need to be evaluated to advance the understanding of the pathogenesis of mesothelioma.

**Acknowledgements**

The authors gratefully acknowledge the assistance of Dr Xin Wang with the irradiation experiments. The advice and encouragement of Dr Curtis C Harris is appreciated as is the editorial assistance of Ms Dorothea Dudek.

### References

BARAK Y AND OREN M. (1992). Enhanced binding of a 95 kDa protein to p53 in cells undergoing p53-mediated growth arrest. *EMBO J.*, **11**, 2115–2121.

BARAK Y, JUVEN T, HAFFNER R AND OREN M. (1993). mdm2 expression is induced by wild type p53 activity. *EMBO J.*, **12**, 461–468.

BARAK Y, GOTTLIEB E, JUVEN-GERSHON T AND OREN M. (1994). Regulation of mdm2 expression by p53: alternative promoters produce transcripts with nonidentical translation potential. *Genes Dev.*, **8**, 1739–1749.

BARRETT JC. (1991). Role of chromosomal mutations in asbestos-induced cell transformation. In *Cellular and Molecular Aspects of Fibre Carcinogenesis. Current Communications in Cell and Molecular Biology 2*. Harris CC, Lechner JF and Brinkley BR. (eds). pp. 27–39. Cold Springer Harbor Laboratory Press: New York.

BENEDICT WF, XU HJ, HU SX AND TAKAHASHI R. (1990). Role of the retinoblastoma gene in the initiation and progression of human cancer. *J. Clin. Invest.*, **85**, 988–993.

BUESO-RAMOS CE, YANG Y, DELEON E, MCCOWN P, STASS SA AND ALBITAR M. (1993). The human MDM-2 oncogene is overexpressed in leukemias. *Blood*, **82**, 2617–2623.

CAHILLY-SNYDER L, YANG-FENG T, FRANCKE U AND GEORGE DL. (1987). Molecular analysis and chromosomal mapping of amplified genes isolated from a transformed mouse 3T3 cell line. *Somat. Cell. Mol. Genet.*, **13**, 235–244.

CARBONE M, PASS HI, RIZZO P, MARINETTI M, DI MUZIO M, MEW DJ, LEVINE AS AND PROCOPIO A. (1994). Simian virus 40-like DNA sequences in human pleural mesothelioma. *Oncogene*, **9**, 1781–1790.

CHEN J, MARECHAL V AND LEVINE AJ. (1993). Mapping of the p53 and mdm-2 interaction domains. *Mol. Cell. Biol.*, **13**, 4107–4114.

CHENG JQ, JHANWAR SC, KLEIN WM, BELL DW, LEE WC, ALTOMARE DA, NOBORI T, OLOPADE OI, BUCKLER AJ AND TESTA JR. (1994). p16 alterations and deletion mapping of 9p21–p22 in malignant mesothelioma. *Cancer Res.*, **54**, 5547–5551.

CHOMCZYNSKI P AND SACCHI N. (1987). Single-step method of RNA isolation by acid guanidinium thiocyanate–phenol–chloroform extraction. *Anal. Biochem.*, **162**, 156–159.

CORDON-CARDO C, LATRES E, DROBNJAK M, OLIVA MR, POLLACK D, WOODRUFF JM, MARECHAL V, CHEN J, BRENNAN MF AND LEVINE AJ. (1994). Molecular abnormalities of mdm2 and p53 genes in adult soft tissue sarcomas. *Cancer Res.*, **54**, 794–799.

CRAIGHEAD JE AND MOSSMAN BT. (1982). The pathogenesis of asbestos-associated diseases. *N. Engl. J. Med.*, **306**, 1446–1455.

DECAPRIO JA, LUDLOW JW, FIGGE J, SHEW JY, HUANG CM, LEE WH, MARSILIO E, PAUCHA E AND LIVINGSTON DM. (1988). SV40 large tumor antigen forms a specific complex with the product of the retinoblastoma susceptibility gene. *Cell*, **54**, 275–283.

DYSON N, HOWLEY PM, MUNGER K AND HARLOW E. (1989). The human papilloma virus-16 E7 oncoprotein is able to bind to the retinoblastoma gene product. *Science*, **243**, 934–937.

EWEN ME, SLUSS HK, SHERR CJ, MATSUSHIME H, KATO J AND LIVINGSTON DM. (1993). Functional interactions of the retinoblastoma protein with mammalian D-type cyclins. *Cell*, **73**, 487–497.

FAKHARZADEH SS, TRUSKO SP AND GEORGE DL. (1991). Tumorigenic potential associated with enhanced expression of a gene that is amplified in a mouse tumor cell line. *EMBO J.*, **10**, 1565–1569.

FARMER G, BARGONETTI J, ZHU H, FRIEDMAN P, PRYWES R AND PRIVES C. (1992). Wild-type p53 activates transcription *in vitro* (see comments). *Nature*, **358**, 83–86.

FINLAY CA. (1993). The mdm-2 oncogene can overcome wild-type p53 suppression of transformed cell growth. *Mol. Cell. Biol.*, **13**, 301–306.

FORT P, MARTY L, PIECHACZYK M, EL SABROUTY S, DANI C, JEANTEUR P AND BLANCHARD JM. (1985). Various rat adult tissues express only one major mRNA species from the glyceraldehyde-3-phosphate-dehydrogenase multigenic family. *Nucleic Acids Res.*, **13**, 1431–1442.

GOODRICH DW AND LEE WH. (1993). Molecular characterization of the retinoblastoma susceptibility gene. *Biochim. Biophys. Acta*, **1155**, 43–61.

GUDAS JM, NGUYEN H, KLEIN RC, KATAYOSE D, SETH P AND COWAN KH. (1995). Differential expression of multiple MDM2 messenger RNAs and proteins in normal ant tumorigenic breast epithelial cells. *Clin. Cancer Res.*, **1**, 71–80.

HAINES DS, LANDERS JE, ENGLE LJ AND GEORGE DL. (1994). Physical and functional interaction between wild-type p53 and mdm2 proteins. *Mol. Cell. Biol.*, **14**, 1171–1178.

HEI TK, PIAO CQ, HE ZY, VANNAIS D AND WALDREN CA. (1992). Chrysotile fibre is a strong mutagen in mammalian cells. *Cancer Res.*, **52**, 6305–6309.

HOLLSTEIN M, SIDRANSKY D, VOGELSTEIN B AND HARRIS CC. (1991). p53 mutations in human cancers. *Science*, **253**, 49–53.

JIANG D, SRINIVASAN A, LOZANO G AND ROBBINS PD. (1993). SV40 T antigen abrogates p53-mediated transcriptional activity. *Oncogene*, **8**, 2805–2812.

KUERBITZ SJ, PLUNKETT BS, WALSH WV AND KASTAN MB. (1992). Wild-type p53 is a cell cycle checkpoint determinant following irradiation. *Proc. Natl Acad. Sci. USA*, **89**, 7491–7495.

LADANYI M, CHA C, LEWIS R, JHANWAR SC, HUVOS AG AND HEALEY JH. (1993). MDM2 gene amplification in metastatic osteosarcoma. *Cancer Res.*, **53**, 16–18.

LANDERS JE, HAINES DS, STRAUSS JF III, AND GEORGE DL. (1994). Enhanced translation: a novel mechanism of mdm2 oncogene overexpression identified in human tumor cells. *Oncogene*, **9**, 2745–2750.

LAVECK MA, SOMERS ANA, MOORE LL, GERWIN BI AND LECHNER JF. (1988). Dissimilar peptide growth factors can induce normal human mesothelial cell multiplication. *In Vitro*, **24**, 1077–1084.

LEACH FS, TOKINO T, MELTZER P, BURRELL M, OLINER JD, SMITH S, HILL DE, SIDRANSKY D, KINZLER KW AND VOGELSTEIN B. (1993). p53 Mutation and MDM2 amplification in human soft tissue sarcomas. *Cancer Res.*, **53**, 2231–2234.

LECHNER JF AND LAVECK MA. (1985). A serum-free method for culturing normal human bronchial epithelial cells at clonal density. *J. Tissue Culture Meth.*, **9**, 43–48.

LECHNER JF, TOKIWA T, LAVECK MA, BENEDICT WF, BANKS-SCHLEGEL SP, YEAGER H, JR., BANERJEE A AND HARRIS CC. (1985). Asbestos-associated chromosomal changes in human mesothelial cells. *Proc. Natl Acad. Sci. USA*, **82**, 3884–3888.

LECHNER JF, LAVECK MA, GERWIN BI AND MATIS EA. (1989). Differential responses to growth factors by normal human mesothelial cultures from individual donors. *J. Cell Physiol.*, **139**, 295–300.

LEVINE AJ, MOMAND J AND FINLAY CA. (1991). The p53 tumour suppressor gene. *Nature*, **351**, 453–456.

LU X AND LANE DP. (1993). Differential induction of transcriptionally active p53 following UV or ionising radiation: defects in chromosome instability syndromes? *Cell*, **75**, 765–778.

MANIATIS, FRITSCH EF AND SAMBROOK J. (1982). *Molecular Cloning: A Laboratory Manual*. Cold Spring Harbour Press: New York.

MARCHETTI A, BUTTITTA F, PELLEGRINI S, MERLO G, CHELLA A, ANGELETTI CA AND BEVILACQUA G. (1995). mdm2 gene amplification and overexpression in non-small cell lung carcinomas with accumulation of the p53 protein in the absence of p53 gene mutations. *Diagn. Mol. Pathol.*, **4**, 93–97.

MARTIN K, TROUCHE D, HAGEMEIER C, SORENSEN TS, LA THANGUE NB AND KOUZARIDES T. (1995). Stimulation of E2F1/DP1 transcriptional activity by MDM2 oncoprotein. *Nature*, **375**, 691–694.

METCALF RA, WELSH JA, BENNETT WP, SEDDON MB, LEHMAN TA, PELIN K, LINNAINMAA K, TAMMILEHTO L, MATTSON K, GERWIN BI AND HARRIS CC. (1992). p53 and Kirsten-ras mutations in human mesothelioma cell lines. *Cancer Res.*, **52**, 2610–2615.

MIETZ JA, UNGER T, HUIBREGTSE JM AND HOWLEY PM. (1992). The transcriptional transactivation function of wild-type p53 is inhibited by SV40 large T-antigen and by HPV-16 E6 oncoprotein. *EMBO J.*, **11**, 5013–5020.

MOMAND J, ZAMBETTI GP, OLSON DC, GEORGE D AND LEVINE AJ. (1992). The mdm-2 oncogene product forms a complex with the p53 protein and inhibits p53-mediated transactivation. *Cell*, **69**, 1237–1245.

MOSSMAN BT, MARSH JP AND SHATOS MA. (1986). Alteration of superoxide dismutase activity in tracheal epithelial cells by asbestos and inhibition of cytotoxicity by antioxidants. *Lab. Invest.*, **54**, 204–212.

OKAMOTO A, DEMETRICK DJ, SPILLARE EA, HAGIWARA K, HUSSAIN SP, BENNETT WP, FORRESTER K, GERWIN B, SERRANO M, BEACH DH AND HARRIS CC. (1994). Mutations and altered expression of genes upregulating the cell cycle G1 checkpoint in human cancer. *Proc. Natl Acad. Sci. USA*, **91**, 11045–11049.

OLINER JD, KINZLER KW, MELTZER PS, GEORGE DL AND VOGELSTEIN B. (1992). Amplification of a gene encoding a p53-associated protein in human sarcomas (see comments). *Nature*, **358**, 80–83.

OLSON DC, MARECHAL V, MOMAND J, CHEN J, ROMOCKI C AND LEVINE AJ. (1993). Identification and characterization of multiple mdm-2 proteins and mdm-2-p53 protein complexes. *Oncogene*, **8**, 2353–2360.

PERRY ME, PIETTE J, ZAWADZKI JA, HARVEY D AND LEVINE AJ. (1993). The mdm-2 gene is induced in response to UV light in a p53-dependent manner. *Proc. Natl Acad. Sci. USA*, **90**, 11623–11627.

PICKSLEY SM AND LANE DP. (1993). The p53-mdm2 autoregulatory feedback loop: a paradigm for the regulation of growth control by p53? *Bioessays*, **15**, 689–690.

PIETENPOL JA AND VOGELSTEIN B. (1993). Tumour suppressor genes. No room at the p53 inn (news; comment). *Nature*, **365**, 17–18.

QUESNEL B, PREUDHOMME C, OSCIER D, LEPELLEY P, COLLYN-D'HOOGHE M, FACON T, ZANDECKI M AND FENAUX P. (1994). Over-expression of the MDM2 gene is found in some cases of haematological malignancies. *Br. J. Haematol.*, **88**, 415–418.

RAMAEL M, SEGERS K AND VANMARCK E. (1994). Differential immunohistochemical staining for retinoblastoma protein with the antibodies C15 and 1F8 in malignant mesothelioma. *Pathol. Res. Pract.*, **190**, 138–141.

RAVETCH JV, SIEBENLIST U, KORSMEYER S, WALDMANN T AND LEDER P. (1981). Structure of the human immunoglobulin mu locus: characterization of embryonic and rearranged J and D genes. *Cell*, **27**, 583–591.

REIFENBERGER G, LIU L, ICHIMURA K, SCHMIDT EE AND COLLINS VP. (1993). Amplification and overexpression of the MDM2 gene in a subset of human malignant gliomas without p53 mutations. *Cancer Res.*, **53**, 2736–2739.

SEGERS K, BACKHOVENS H, SINGH SK, DE VOECHT J, RAMAEL M, VAN BROECKHOVEN C AND VAN MARCK E. (1995). Immunoreactivity for p53 and mdm2 and the detection of p53 mutations in human malignant mesothelioma. *Virchows. Arch.*, **427**, 431–436.

VAN DER MEEREN A, SEDDON MB, KISPERT J, HARRIS CC AND GERWIN BI. (1993). Lack of expression of the retinoblastoma gene is not frequently involved in the genesis of human mesothelioma. *Eur. Resp. Rev.*, **3**, 177–179.

VOGELSTEIN B AND KINZLER KW. (1992). p53 function and dysfunction. *Cell*, **70**, 523–526.

WAGNER JC AND BERRY G. (1969). Mesotheliomas in rats following inoculation with asbestos. *Br. J. Cancer*, **23**, 567–581.

WAGNER JC, SLEGGS CA AND MARCHAND P. (1960). Diffuse pleural mesothelioma and asbestos exposure in the North Western Cape Province. *Br. J. Ind. Med.*, **17**, 260–271.

WANG XW, VERMEULEN W, COURSEN JD, GIBSON M, LUPOLD SE, FORRESTER K, XU G, ELMORE L, YEH H, HOEIJMAKERS JHJ AND HARRIS CC. (1996). The XPB and XPD helicases are components of the p53-mediated apoptosis pathway. *Genes Dev.*, **10**, 1219–1232.

WHYTE P, BUCHKOVICH KJ, HOROWITZ JM, FRIEND SH, RAYBUCK M, WEINBERG RA AND HARLOW E. (1988). Association between an oncogene and an anti-oncogene: the adenovirus E1A proteins bind to the retinoblastoma gene product. *Nature*, **334**, 124–129.

WU X, BAYLE JH, OLSON D AND LEVINE AJ. (1993). The p53-mdm-2 autoregulatory feedback loop. *Genes Dev.*, **7**, 1126–1132.

XIAO S, LI D, CORSON JM, VIJG J AND FLETCHER JA. (1995). Codeletion of *p15* and *p16* genes in primary non-small cell lung carcinoma. *Cancer Res.*, **55**, 2968–2971.

XIAO ZX, CHEN J, LEVINE AJ, MODJTAHEDI N, XING J, SELLERS WR AND LIVINGSTON DM. (1995). Interaction between the retinoblastoma protein and the oncoprotein MDM2. *Nature*, **375**, 694–698.

YEW PR AND BERK AJ. (1992). Inhibition of p53 transactivation required for transformation by adenovirus early 1B protein. *Nature*, **357**, 82–85.

ZAUBERMAN A, BARAK Y, RAGIMOV N, LEVY N AND OREN M. (1993). Sequence-specific DNA binding by p53: identification of target sites and lack of binding to p53–MDM2 complexes. *EMBO J.*, **12**, 2799–2808.

ZAUBERMAN A, FLUSBERG D, HAUPT Y, BARAK Y AND OREN M. (1995). A functional p53-responsive intronic promoter is contained within the human *mdm2* gene. *Nucleic Acids Res.*, **23**, 2584–2592.

ZHOU M, YEAGER AM, SMITH SD AND FINDLEY HW. (1995). Overexpression of the MDM2 gene by childhood acute lymphoblastic leukemia cells expressing the wild-type p53 gene. *Blood*, **85**, 1608–1614.